# Foreign Bodies in the Meatus

**Published:** 1864-09

**Authors:** Jerome M. Foster


BUFFALO

### Medical and Surgical Journal.

VOL. IV. SEPTEMBER, 1864. No. 2.

ART. I.—*Foreign Bodies in the Meatus.*—By Jerome M. Foster, M. D.

July 24th, 1864, a boy, J. S., twelve years of age, was brought to me, who complained of great pain in the right ear accompanied with dizziness. During the two days previous to consulting me he had been very feverish and vomited occasionally—to such a painful extent on the day that I saw him that he was greatly exhausted. His parents informed me that four years previous he had carelessly dropped a grain of corn into the ear where it had remained ever since, though repeated efforts had been made to remove it by different physicians—in fact, so much inflammation and pain always followed their efforts that it was with difficulty I induced the lad to an examination of the ear.

The first examination of the case developed great inflammation of the dermis, while the tragus was so much swollen as to render it difficult to introduce the speculum. There was an absence of cerumen except where I discovered the grain of corn, which was impacted or adhering very firmly to the anterior wall of the meatus and in contact with the membrana tympani; it was imbedded in a mass of hardened wax, the color of which was almost black. The membrana tympani was almost entirely hid from view. The hearing was almost gone—recognizing the tick of a watch only when pressed closed upon the auricle. My first intention was to remove the mass with the syringe but found it required so much force to make it yield that I adopted the following course:

I applied four Swedish leeches, after which I ordered him to be kept very quiet and upon low diet until the following day. I also directed the ear to be filled with warm olive oil every three or four hours, at the same time giving the patient a liberal cathartic. The next (second) day I found the inflammation much reduced and the fever, generally, subdued. Examining the meatus I found the mass of wax softer, and preparing an injection of soda-bi-carb. ʒ iv, to aqua ferv: ℥ ii, (the precise warmth of the liquid was 103° Fahrnheit,) I resumed the syringe, directing the liquid along the posterior wall of the meatus. I began the syringing gently and increased the force gradually until at the fifth injection, the patient exclaimed that he felt something break—two more injections brought the mass to the orifice which I removed with the forceps—requiring some little force owing to the inflammation. I again examined the meatus and membrana tympani—the latter was concave, red and the vessels of the dermoid layer distended.

I now prescribed the following :

℞ Sub. Mur. Hydrarg, grs. vi.
Pulv. opii, gr. ss. M.*

to soothe the nervous system and prevent re-action. I ordered him to call again the next day. In the meantime I examined the mass I had taken from his ear. It was a small grain of corn almost round, and was in almost perfect state of preservation, imbedded in a thick coat of hardened wax, together weighing grs. xvii; the grain of corn after being cleansed weighed grs. vii.

That a foreign body could so long remain thus situated, in direct contact with the membrana tympani, without causing pain or inflammation, may at first glance seem strange, but experience teaches that *smooth* foreign bodies may continue in the meatus for many months, even years, with impunity, with only the effect of producing a dullness of hearing by simple obstruction to sound; the hearing being at once restored by removal of the substance. With a hard rough substance it is quite the reverse and immediate removal is imperative.

The acute inflammation produced in the case I here quote from my practice was the result of a recent endeavor to "pick out" the grain with a knitting needle.

The dizziness was produced by the grain being worked too far inwards till it pressed against the membrana tympani, forcing the chain of ossicles inwards, thus pressing the stapes towards or against the contents of the vestibule. The coughing and vomiting I traced to irritation of the auricular branch of the pneumo-gastric nerve. Very often an accumulation of har-

* Would not the opium have soothed the system, without the mercury equally well, and the inflammation have subsided upon removal of the cause?—Ed.

dened wax will be loosened or its position changed by deglutition, producing like symptoms.

The third day the patient again came to my office. I found the fever had entirely disappeared, and the local inflammation surprisingly reduced. The suppuration that I had expected to supervene did not appear. The case was progressing as favorably as I could expect, and I desired him to see me again in three or four days, when, upon examination, I found the meatus normal, and membrana tympani almost so, being slightly concave. I then introduced the Eustachian catheter and sent a stream of warm air through the Eustachian tube into the cavity of the tympanum. In a few seconds the patient noticed a loud crack, and heard immediately. The ear was then normal and both ears heard the ticking of a watch at three yards distance. I have seen the boy occasionally since, and the ear preserves its integrity.

Carefully recorded data are always interesting to the thoughtful practitioner, as from such are born many excellent suggestions in general practice; for this reason I have been very minute.

The syringe alone, in even seemingly difficult cases, should be the *only* instrument used for the dislodgment of foreign bodies from the meatus. *I know of no exception to this rule*, for by employing forceps, wires, or other implements, the substance is impelled inwards, as it was in this case, till it presses against the membrana tympani, in the majority of cases producing serious consequences. I have met with many cases where I found the most lamentable results follow the too free use of probes for removing extraneous substances from the meatus. One is now under my treatment, a farmer, sixty-four years of age, whose right ear was injured about eleven years ago by a physician injudiciously using a curved instrument to remove a bean from the meatus. The result in this case is the entire destruction of the sense of hearing in that ear, the *portio dura nerve* being paralysed. This case, however, will be the subject of another article.

Where the case is of recent occurrence, the use of the syringe even should be limited. If, after the inflammation has been reduced, there be no cerumen observable, the greatest care should be observable in prosecuting the examination of the membrana tympani, that the condition of it be well known as in many cases where there is no cerumen the too forcible use of the syringe may rupture the epidermis which is often the only lamina left of the membrana tympani. The surgeon may, however, make himself almost certain of its condition by the use of a magnifying lens in conjunction with the speculum auris to discover the existence of the fibres composing the radiate fibrous lamina.

